# Editorial: Cardiac Tumors: A Challenge in Diagnosis and Therapeutic Approach

**DOI:** 10.3389/fcvm.2022.951357

**Published:** 2022-06-28

**Authors:** Chiara Lestuzzi

**Affiliations:** Department of Cardiology, Santa Maria degli Angeli Hospital Pordenone, Pordenone, Italy

**Keywords:** cardiac tumors (MeSH), cardio-oncology, cardiac malignancy, cardiac malignant tumors, cardiac tumor prognosis, cardiac tumor treatment, cardiac tumor diagnosis

This topic collected a series of cases of cardiac tumors highlighting the difficulties in approaching a rare disease.

Most of the heart tumors are first detected at echocardiography, either performed looking for the cause of cardiac symptoms or during a routine examination, but a multimodality imaging approach, including Computed Tomography (CT), Magnetic Resonance Imaging (MRI) and nuclear medicine is important in the differential diagnosis and in planning the most appropriate treatment.

The benign tumors have a better prognosis of the rarer malignant tumors, and the prognosis of malignant tumors largely depends by the complete resection (R0, i.e., without any even microscopic infiltration of the resection margins) (1–5).

Surgery is the mainstay of treatment for cardiac tumors, but the surgical approach varies according to the site, size and relationship with the cardiac structures, and a neoadiuvant (preoperative) chemotherapy may be necessary to improve the resectability (6).

The cases collected in this topic are good examples of these concepts.

In the case described by Ahmad et al. the problem of differential diagnosis between tumor and infective endocarditis had arisen; in this particular case the use of Positron Emission Tomography (FDG-PET) ruled out inflammation and -at the same time- the presence of a distant cancer which might be the source of a metastatic lesion.

The exact definition of the size and implant of the tumor, and of its relationship with the surrounding structures is essential in planning the best surgical approach. One case report in this series shows the importance of 3D CT scan in planning the surgical approach Song et al.. In another case, regarding a complex left atrial tumor, 3D printed reconstruction of the mass was helpful in planning surgery Zhou et al.. This case also highlights the advantages of cardiac autotransplantation in approaching the left atrial tumors, following the method currently used by the team of Reardon at the Methodist Hospital of Houston (7).

The paper of Li et al. describes a case of a tumor (Gastrointestinal Neuroectodermal Neural Tumor or GNET) which is very rare in general, and exceptional in the heart as the primary site. The role of pathology is of utmost importance in defining the primary cardiac tumors and this particular case highlights the complexity of the diagnosis and the essential role of immunohistochemistry. This case also confirms the well-known concept that an incomplete resection of a malignant tumor -if not associated to chemo- or radiotherapy- leads to an early local relapse. The problem of incomplete surgery is less relevant in benign tumors, which may re-grow only slowly, or even not at all, as happened in another case described by Song et al..

Cardiac myxomas are usually sporadic, with a good prognosis after resection. The patients with PRKARIA mutation are, however, at risk of relapse. Ling-Yun describes a very peculiar case of a patient who presented first with multiple sites mixomas and who developed a symptomatic bi-cameral relapse just 1 year after the routine echocardiographis follow-up Kong et al.. This case suggests that in patients with Carney complex a strict follow-up is necessary.

The last paper in this series does not regard a cardiac tumor but rather the problem of differential diagnosis in cardiac amyloidosis; the patient described had multiple comorbidities, an intriguing clinical presentation and eventually a plasma cell disorder, requiring antineoplastic treatment with bortezomib, cyclophosphamide and dexamethasone Lv et al..

In conclusion, this case series confirms that -in presence of a suspected cardiac tumor- a multidisciplinary cardiac tumor team, including cardio-oncologists, imaging experts, cardiac surgeons and oncologist is necessary to manage an often challenging patient (8).

## Author Contributions

The author confirms being the sole contributor of this work and has approved it for publication.

## Conflict of Interest

The author declares that the research was conducted in the absence of any commercial or financial relationships that could be construed as a potential conflict of interest.

## Publisher's Note

All claims expressed in this article are solely those of the authors and do not necessarily represent those of their affiliated organizations, or those of the publisher, the editors and the reviewers. Any product that may be evaluated in this article, or claim that may be made by its manufacturer, is not guaranteed or endorsed by the publisher.
